# Online Search Trends Related to Bariatric Surgery and Their Relationship with Utilization in Australia

**DOI:** 10.1007/s11695-024-07457-1

**Published:** 2024-08-14

**Authors:** Roy Huynh, Vyshi Satchithanandha, Jin-Soo Park, Doruk Seyfi, David Joseph

**Affiliations:** 1 Department of Upper Gastrointestinal Surgery, Royal Prince Alfred Hospital, Level 7 – Main Clinical Building, 50 Missenden Road, Camperdown, NSW 2050 Australia; 2 Faculty of Medicine, UNSW Sydney, Kensington, NSW 2050 Australia; 3 Faculty of Medicine, University of Sydney, Camperdown, NSW 2050 Australia

**Keywords:** Bariatric surgery, Obesity, Internet, Online information

## Abstract

**Purpose:**

There is an abundance of online information related to bariatric surgery. Patients may prefer a specific type of bariatric surgery based on what they read online. The primary aim of this study was to determine online search trends in bariatric surgery over time in Australia and worldwide. The secondary aim was to establish a relationship between public online search activity and the types of bariatric surgery performed in Australia.

**Materials and Method:**

The terms “adjustable gastric band,” “sleeve gastrectomy,” and “gastric bypass surgery” were submitted for search volume analysis in Australia and worldwide using the Google Trends “Topic” search function. This was compared alongside the numbers of gastric bandings, sleeve gastrectomies, and gastric bypass surgeries performed in Australia over time to determine if there was a relationship between the two.

**Results:**

Search trends for “adjustable gastric band” and “sleeve gastrectomy” in Australia were similar to trends seen worldwide. However, search trends for “gastric bypass surgery” differ between Australia and the rest of the world. It took at least a year for online searches to reflect the higher number of sleeve gastrectomies performed relative to gastric bandings. There was a lag time of over four years before online searches reflected the higher number of gastric bypass surgery performed compared to gastric banding.

**Conclusion:**

Search interests in Australia and worldwide were similar for gastric banding and sleeve gastrectomy but different for gastric bypass surgery. Online search activity did not have a significant association with the types of bariatric surgery being performed in Australia.

**Graphical Abstract:**

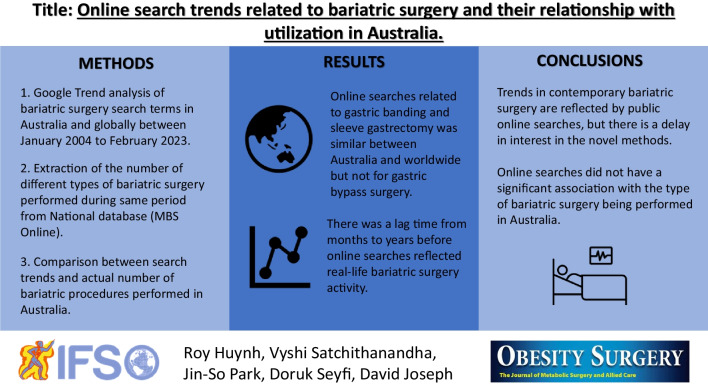

## Introduction

Bariatric surgery is currently the most successful modality for weight loss. Most obese patients undergoing bariatric surgery can achieve approximately 25–30% weight loss [1, 2]. It is currently the only successful long-term weight loss modality that results in remission of obesity-related diseases such as diabetes mellitus, obstructive sleep apnea, and hypercholesterolemia [3–5]. The success of bariatric surgery has captured significant public interest with studies demonstrating increased online interest in bariatric surgery over time [6]. Patients considering bariatric surgery will often access information from the internet about the different types of bariatric surgery prior to seeing a surgeon [7]. There exists a plethora of online forums, social media groups, and medical websites providing information about bariatric surgery [6–10]. Although the accuracy and readability of online information are questionable, the internet remains a popular source of information for patients seeking weight loss and bariatric surgery [11].

It has become increasingly common for patients to make weight loss decisions based on online information. The internet age has allowed easy accessibility to information related to weight loss [12]. One study demonstrated 25% of patients pursuing bariatric surgery did so after researching weight loss online [13]. Patients may prefer a specific type of bariatric surgery based on information they read on the internet. However, this preference may not be evidence-based, nor be in the patient’s best interest [14, 15]. Rozier and colleagues showed that patients consider multiple factors when deciding on a particular type of bariatric surgery. The choice of procedure was most strongly governed by cost, total weight loss, and resolution of obesity-related comorbidities [16]. The oversimplification of these factors on the internet can misguide patients into preferencing certain types of bariatric procedures that may not be appropriate based on their individual circumstances.

Patients can have a significant impact on a surgeon’s decision-making process. Multiple studies have shown that while doctors believe they are objective when making medical decisions, they are undoubtedly influenced by patients’ preference [17]. This is particularly true for bariatric surgery, which is extensively promoted on online outlets such as social media [18]. In addition, patients’ perspectives are becoming more influential in clinical-decision making [19]. There have been significant changes in the types of bariatric surgery performed in Australia over time. Most notably, laparoscopic sleeve gastrectomy has replaced laparoscopic adjustable gastric banding as the most popular primary bariatric procedure [20]. More recently, gastric bypass surgery has become increasingly favored due to its superior weight loss profile [21, 22]. To what extent the internet has played in these changes remains unclear. Studies have shown that 47% of patients will discuss information they found on the internet with their clinician [23]. Patients exposed to online information can expressed preference for a particular type of weight loss surgery, which can have an influence on the decision-making process of bariatric surgeons [24].

The primary aim of this study was to establish online search trends over time related to gastric banding, sleeve gastrectomy, and gastric bypass surgery in Australia and worldwide. The secondary aim was to determine whether there exists a relationship between online search trends and the types of bariatric surgery performed in Australia.

## Methods

### Google Search Trend

Google Trends was used to establish the online search trend of bariatric surgery in Australia and worldwide from January 2004 (when Google Trends was implemented) to February 2023. This method of gauging online search interest related to bariatric surgery has been used in multiple other studies [25–27]. Google Trends provides an unbiased analysis of search terms on Google.com over time. The search engine Google.com encompasses 84–90% market share of all search engines and is therefore an accurate way of gauging online interest in bariatric surgery [28]. Google Trends generates graphs showing relative search interest of a search term over time. The vertical axis of the graphs consists of a score between 0 to 100, with 0 indicating no search interest and 100 being peak search interest. Any number in between represents a percentage of peak search interest at a particular time.

The terms “adjustable gastric band,” “sleeve gastrectomy,” and “gastric bypass surgery” were submitted to Google Trends using the “Topic” search function, which enables all synonyms, misspellings, and semantic variations related to the terms to be captured. Google Trends enabled the search interest of each bariatric surgery term in Australia to be compared to the rest of the world. The relative search interest of each bariatric surgery term was compared to the actual number of procedures performed in Australia over time to determine if there was any association between public online interest and types of bariatric procedures performed [29].

### Bariatric Surgery Data Source

The number of gastric bandings, sleeve gastrectomies, and gastric bypass surgeries performed in Australia was obtained using publicly available data from the Australian Government’s Services Australia website. This website generates reports on medical procedures listed on the Medicare Benefit Schedule (MBS). The MBS contains procedures subsidized by the Australian healthcare system [29]. Each procedure is allocated a unique item number. Surgeons use these item numbers to claim financial rebates on procedures performed. This model of healthcare funding applies to both the public and private healthcare system in Australia. Surgeons can charge patients a “gap” fee on top of the rebate they received from the MBS to cover additional practice costs.

The MBS item numbers for gastric banding (31,569), sleeve gastrectomy (31,575), and gastric bypass (31,572 and 31,581) were used to generate reports on the number of each procedure performed every month in Australia from July 2013 to February 2023. Prior to July 2013, there was only a single item number (30,511) that covered all bariatric surgeries and did not distinguish between gastric banding, sleeve gastrectomy, and gastric bypass. It was also not possible to differentiate between the types of gastric bypass surgeries (e.g. single anastomosis gastric bypass, Roux-en-Y gastric bypass) based on the item numbers as there was significant variability between what different surgeons would submit.

### Study Outcomes

The primary aim of this study was to determine public online interest in gastric banding, sleeve gastrectomy, and gastric bypass over time in Australia and worldwide. The secondary aim was to determine whether there was an association between online search interest and the types of bariatric procedures performed in Australia.

### Data Analysis

Data analysis was performed using Microsoft Excel v16.0. Google Trends produced graphs of the relative search interest over time in Australia and worldwide. The number of gastric bandings, sleeve gastrectomies, and gastric bypass surgeries performed each month were plotted on linear graphs. Potential relationships between online search trends and bariatric procedures performed were determined from analysis of the graphs.

### Ethics

This study was performed using publicly available data from Services Australia and Google Trends. As such, it was deemed exempt from the Institutional Review Board at our institution. The patient data collected were all de-identified and did not require informed consent as it uses publicly available data.

## Results

### Online Searches Related to Bariatric Surgery in Australia

**Fig. 1:**
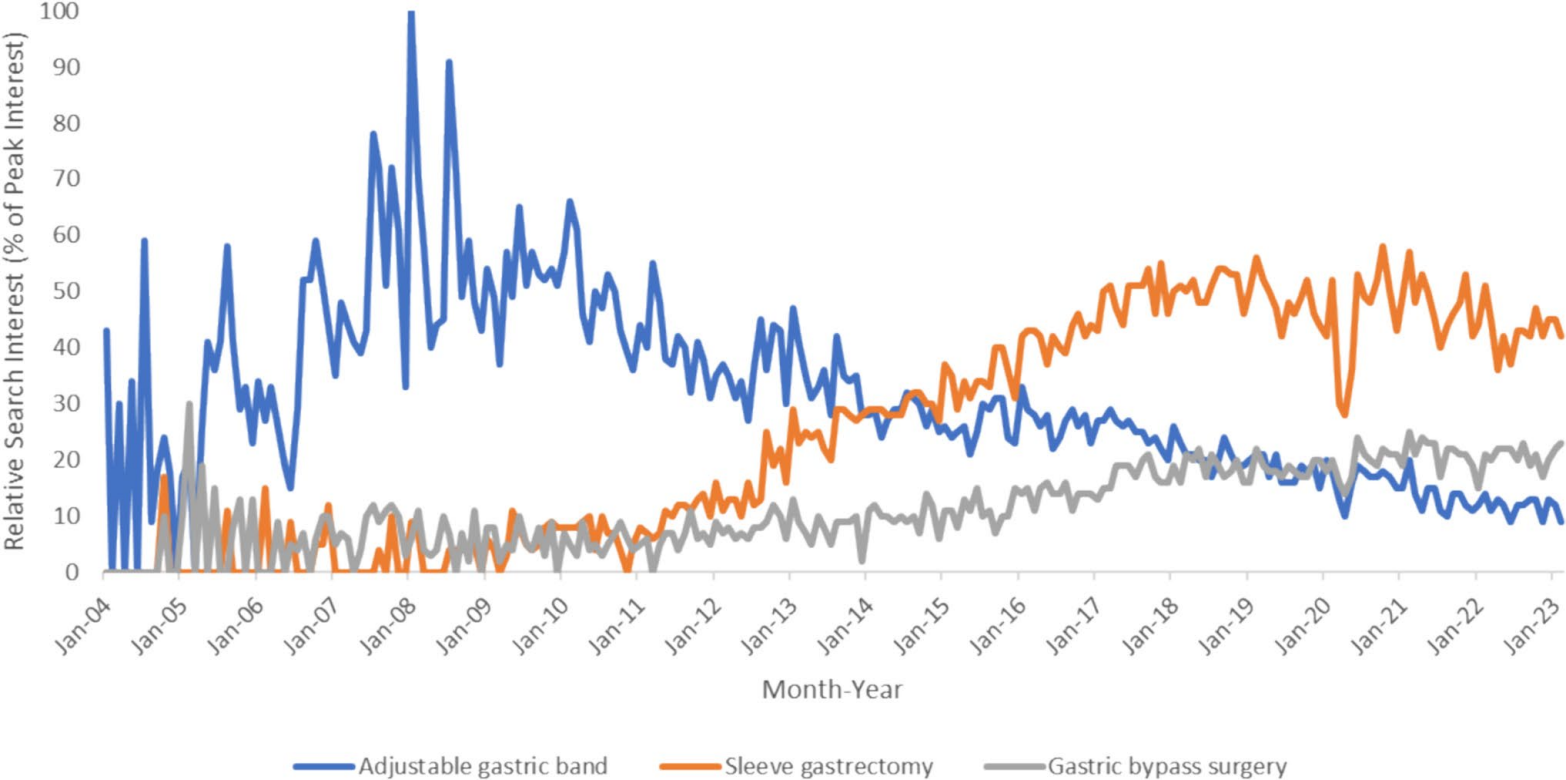
Australia’s search interest of bariatric terms, January 2004–February 2023

Figure 1 depicts the Google trends of bariatric search terms in Australia from January 2004 to February 2023. The number of Google searches related to gastric banding peaked in January 2008 before undergoing a steady decline. Conversely, searches related to sleeve gastrectomy has increased with time. January 2014 was the first month when the number of Google searches related to sleeve gastrectomy became higher than that of gastric banding. The number of searches for both terms was then comparable until August 2014 when searches related to sleeve gastrectomy remained definitively higher than that of gastric banding.

Google searches related to gastric bypass surgery in Australia have fluctuated over time but remained relatively steady until mid-2014 when it started to increase. Searches related to gastric bypass surgery and sleeve gastrectomy were comparable until October 2011 when searches related to sleeve gastrectomy became consistently higher than that of gastric bypass surgery. Searches related to gastric bypass surgery definitively overtook that of gastric banding in February 2020.

There was a notable decrease in searches related to all three bariatric procedures in Australia around April 2020 reflecting the shift in online interest towards the COVID-19 pandemic.

### Online Searches Related to Bariatric Surgery Worldwide

**Fig. 2:**
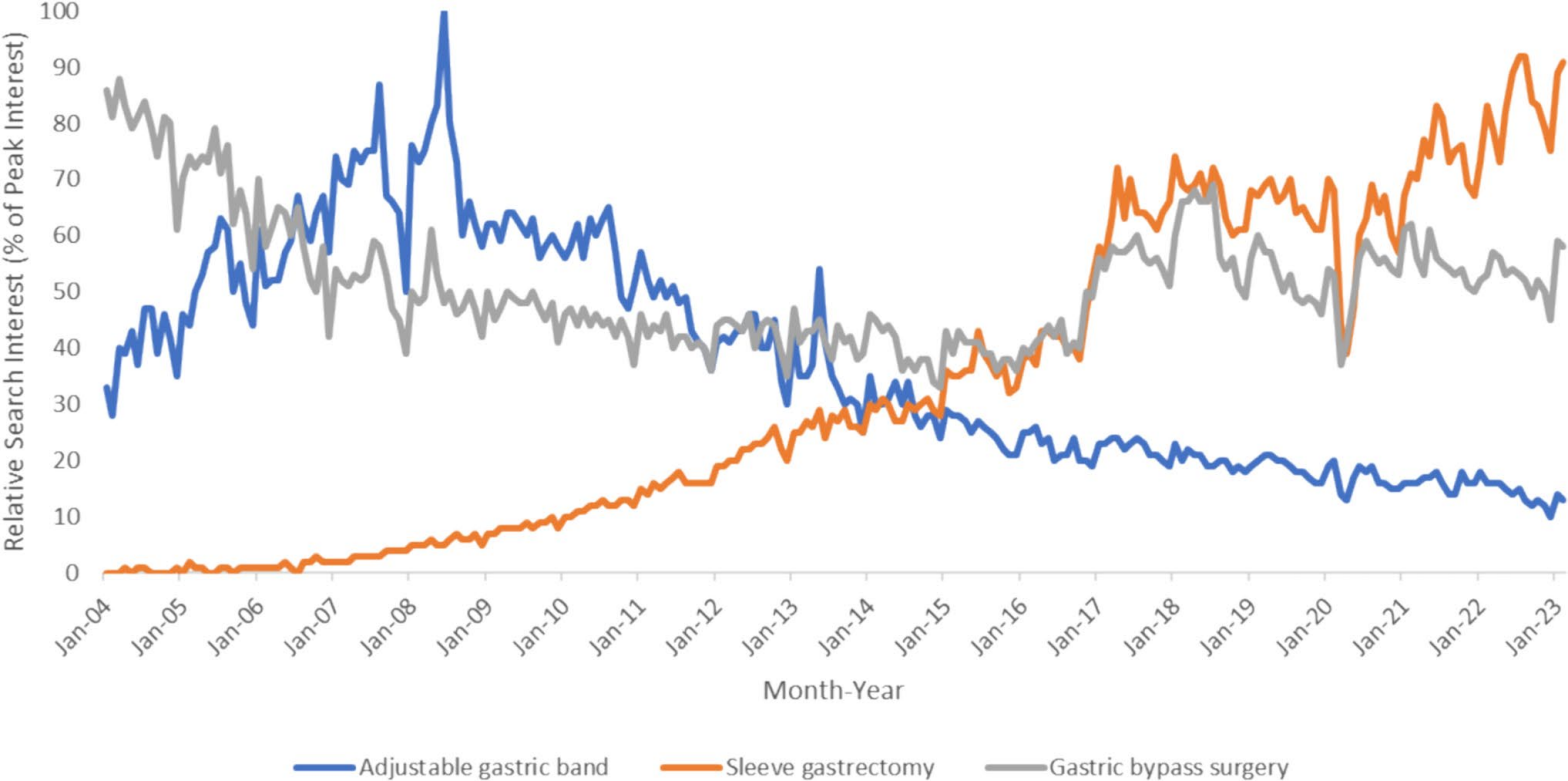
Worldwide search interest of bariatric terms, January 2004–February 2023

Figure 2 depicts the Google trends of bariatric search terms worldwide from January 2004 to February 2023. Global searches related to sleeve gastrectomy and gastric banding were similar to Australia. Global searches related to gastric banding peaked in June 2008 before declining. The number of worldwide searches related to sleeve gastrectomy has steadily increased over time. March 2014 marked the first time when worldwide searches related to sleeve gastrectomy were higher than that of gastric banding. Searches of both terms were then comparable until August 2014, when searches related to sleeve gastrectomy become permanently higher than that of gastric banding.

Worldwide searches related to gastric bypass surgery differ to that seen in Australia. Searches related to gastric bypass surgery were initially higher than searches related to both gastric banding and sleeve gastrectomy. There has been a decline in search popularity of gastric bypass surgery until early 2008 when searches of the term remained relatively steady. On June 2015, searches related to sleeve gastrectomy overtook that of gastric bypass surgery for the first time ever. On December 2016, searches related to sleeve gastrectomy remained permanently higher than gastric bypass surgery. Searches related to gastric bypass surgery became lower than that of gastric banding in July 2006 and remained so until October 2011, when both search terms became comparable. Searches related to gastric bypass surgery permanently became higher than gastric banding after July 2013.

Like Australia, there was a global decrease in searches related to all three bariatric procedures in April 2020.

### Bariatric Surgery Activity in Australia Over Time

**Fig. 3:**
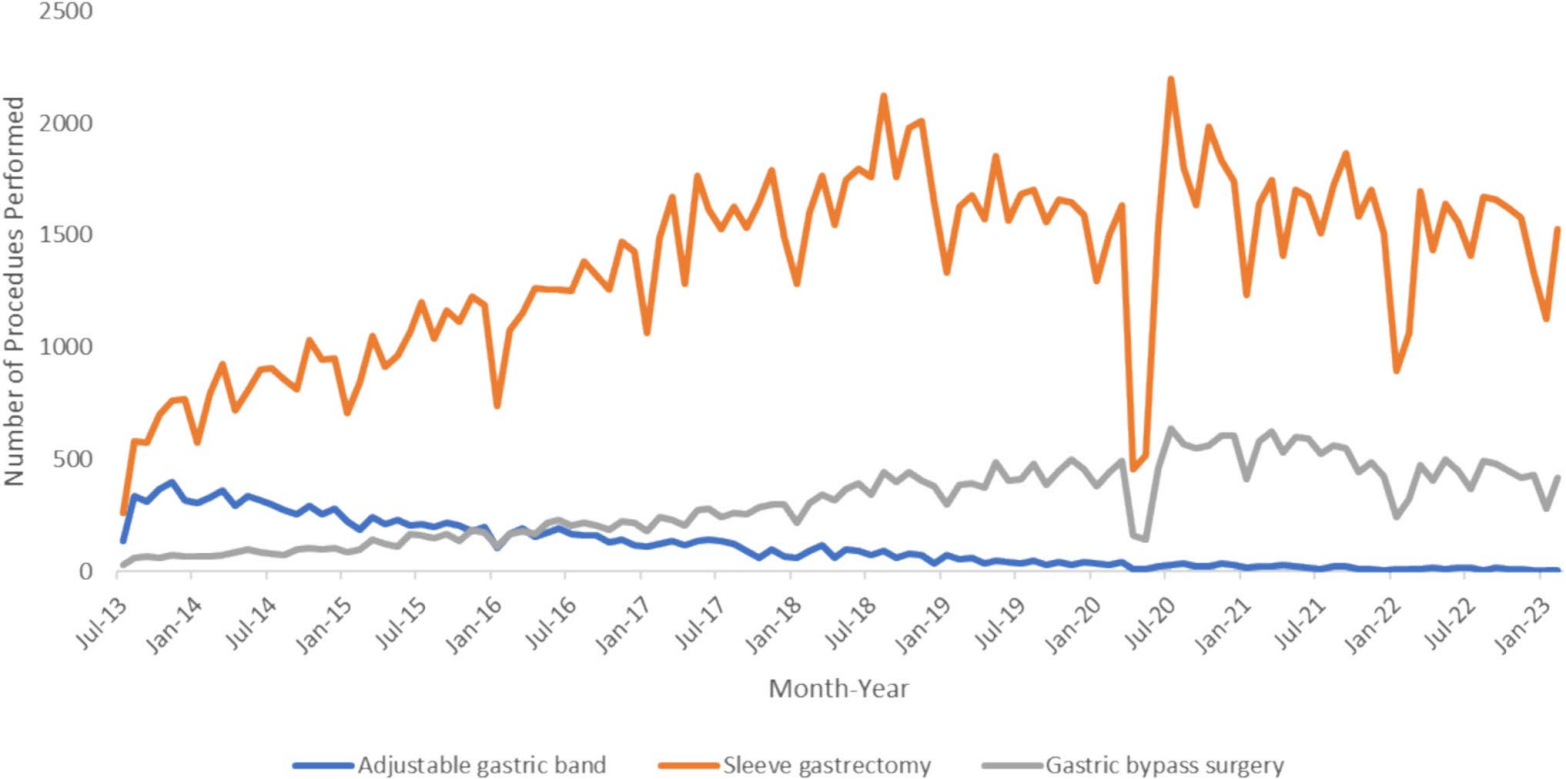
Bariatric procedures performed in Australia, July 2013–February 2023

Figure 3 outlines the number of different bariatric surgeries performed in Australia from July 2013 to February 2023. The number of sleeve gastrectomies performed in July 2013 was higher than the number of gastric bandings and gastric bypass surgeries performed. Initially, more gastric bandings were performed than gastric bypass surgery. This changed in November 2015 when the number of gastric bypass surgeries performed became higher than the number of gastric bandings performed for the first time ever. The number of gastric bandings and gastric bypass surgery performed was then comparable until April 2016 when the number of gastric bypass surgery performed became permanently higher than the number of gastric bandings performed.

### Association Between Online Search Trends and Bariatric Activity in Australia

Changes in online search activity appear to lag behind real life events. Our analysis showed the number of sleeve gastrectomies performed in Australia was higher than the number of gastric bandings performed in July 2013. However, search volumes related to sleeve gastrectomies only overtook that of gastric bandings permanently in August 2014. Similarly, the number of gastric bypass surgery performed in Australia became higher than gastric bandings permanently in April 2016. It took until February 2020 for search volumes of gastric bypass surgery to overtake that of gastric banding.

## Discussion

Our results demonstrated that search volumes of “adjustable gastric band” and “sleeve gastrectomy” were similar in Australia and worldwide. In both cases, there has been a steady decline in searches related to gastric banding and an increase in searches related to sleeve gastrectomy. There was a two-month gap between Australia and worldwide of when search volumes related to sleeve gastrectomy first overtook that of gastric banding. Online searches related to sleeve gastrectomy in Australia and worldwide permanently overtook that of gastric banding during the same month in August 2014. The similar online search activity between Australia and worldwide related to gastric banding and sleeve gastrectomy is unsurprising given the popularity of these two bariatric procedures. Furthermore, availability of online information nowadays means that people, regardless of their country of residence, will have similar accessibility [30]. Our findings mirror that of other studies, which also showed similar search activity related to gastric banding and sleeve gastrectomy in their country compared to the rest of the world [25, 26].

Interestingly, our study revealed searches related to gastric bypass surgery was significantly different between Australia and worldwide. It appears that searches in Australia related to gastric bypass surgery relative to other bariatric procedures has only steadily become popular recently while this has always been the case worldwide. This difference may be due to significant variations in public interest of gastric bypass surgery between Australia and the rest of the world. Indeed, published data suggest that countries such as Belgium perform more gastric bypass surgery for weight loss than any other procedures, whereas sleeve gastrectomy remains the most performed procedure for weight loss in Australia [31]. It may also be possible that not all searches of gastric bypass surgery are related to weight loss. The “Topic” search function was used in our study to ensure that all queries of gastric bypass surgery are captured regardless of whether they are related to weight loss or not. Being unable to distinguish internet users’ intent prevented the accurate comparison of search activity of gastric bypass surgery for weight loss between Australia and the rest of the world.

The Internet is a major source of Information for patients seeking bariatric surgery [9–15]. Over-reliance of online information may prompt certain patients to seek out a particular type of bariatric surgery that may not be in their best interest. Prior to this study, it was unclear whether the internet played a significant role in governing the types of bariatric surgery that were been performed. Kaminski and colleagues utilized Google Trends to analyze online interest of bariatric procedures and other weight loss approaches [32]. Their findings on worldwide search trends were congruent to ours, but they did not correlate this with the types of procedures that were been performed. Our study demonstrated that there was a lag time before online searches reflected real-life bariatric surgery activity in Australia. It took at least a year for online search volumes in Australia to reflect the higher number of sleeve gastrectomies being performed compared to gastric bandings. It took over four years for online searches in Australia to reflect that the number of gastric bypass surgeries had exceeded the number of gastric bandings being performed. Our findings suggested that public online interest did not have a significant role in governing the types of bariatric surgery being performed as online search activity appears to reflect changes in surgical practice after months to years.

Our study was limited by the function of Google Trends. Information related to the types of users that were searching for information on bariatric surgery was unable to be determined. This prevented deeper analysis into the role that the internet has in influencing patients’ decision in choosing a particular type of bariatric surgery. Furthermore, the Google Trends Topic search function data does not allow specific search terms to be identified. Rather, it pools together search terms that share the same concept or entity. Nonetheless, Google Trends is still a reliable tool in analyzing public perception of bariatric surgery and has been used in multiple studies. Google Trends also produces relative search volume data rather than outputting the actual number of hits for each search term. This limits our analytical ability but was adequate for this study as our intention was to compare the search volumes of different bariatric surgeries. Google trends allow association items to be identified and this could have been explored in our study. However, this was not considered during the design phase of the study. Future studies could consider how media publications on bariatric surgery can impact public online search activity. The retrospective nature of this study is also another limitation. Item numbers submitted by surgeons may be prone to collecting error. Furthermore, analysis of the types of bariatric procedures performed in Australia was restricted to post-July 2013 as prior to his, there was no item numbers that could distinguish the different types of bariatric procedure. The MBS data also does not distinguish between bariatric procedures that were performed in private or public hospitals. This prevented assessing whether public search interest had a bigger impact in the private or public sector.

## Conclusion

Trends in contemporary bariatric surgery practice are reflected by public online searches, but there is a delay in interest in the novel procedures. Online searches related to gastric banding and sleeve gastrectomy was similar between Australia and worldwide, but searches related to gastric bypass surgery was not. Online search activity did not have a significant association with the types of bariatric surgery being performed in Australia.

## Data Availability

Statement stating that all the data are publicly available has been included. The methods described where the data can be accessed online.
